# Crystal structure of 1-[(1*S*,2*R*)-2-hydroxy-1-methyl-2-phenyl­eth­yl]pyrrolidinium 2-amino-5-chloro­benzoate

**DOI:** 10.1107/S2056989015013389

**Published:** 2015-07-17

**Authors:** Yunli Li, Zhanjun Li, Yanjie Hu, Wen Li

**Affiliations:** a School of Chemical Engineering and Energy, Zhengzhou University, Zhengzhou 450001, People’s Republic of China; b Institute of Pharmaceutical Research, Zhengzhou University, Zhengzhou 450001, People’s Republic of China, School of Pharmacy, Zhengzhou University, Zhengzhou 450001, People’s Republic of China

**Keywords:** crystal structure, 2-amino-5-chloro­benzoate anion, 1-[(1*S*,2*R*)-2-Hy­droxy-1-methyl-2-phenyl­eth­yl]pyrrolidinium cation, hydrogen bonding

## Abstract

In the cation of the title mol­ecular salt, C_13_H_20_NO^+^·C_7_H_5_ClNO_2_^−^, the five-membered ring adopts a twisted conformation about one of the C—N bonds. The exocyclic N—C bond has an equatorial orientation. The dihedral angle between the five-membered ring (all atoms) and the benzene ring is 76.56 (19)°. In the anion, the dihedral angle between the carboxyl­ate group and the benzene ring is 18.57 (14)°, and an intra­molecular N—H⋯O hydrogen bond closes an *S*(6) ring. In the crystal, the components are linked by O—H⋯O and N—H⋯O hydrogen bonds, generating [100] chains.

## Related literature   

For the crystal structures of related compounds, see: Pennemann *et al.* (2000 ▸); Sugiyama *et al.* (2002 ▸); Ishida *et al.* (2001 ▸). For bond-length data of chloro­benzoate derivatives, see: Arora & Pant (1969 ▸). For applications of the title compound and further synthetic details, see: Kanizsai *et al.* (2006 ▸); Rzaczynska *et al.* (2000 ▸).
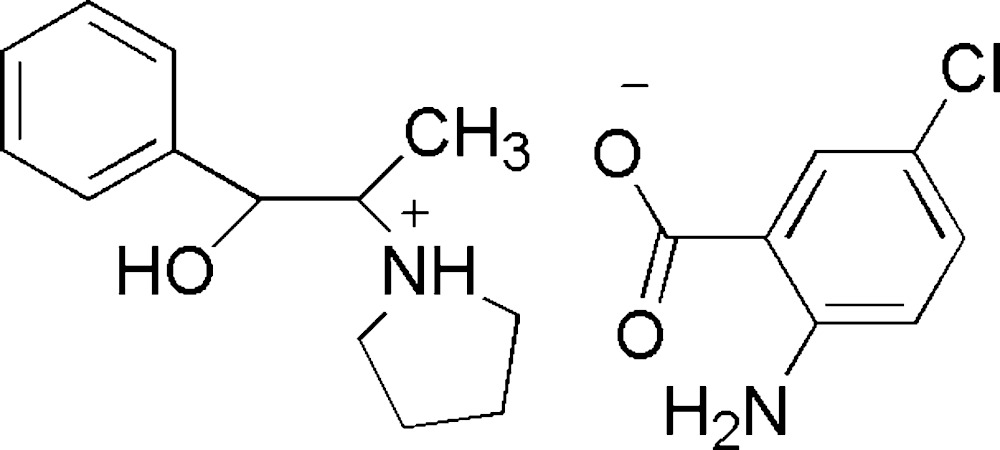


## Experimental   

### Crystal data  

C_13_H_20_NO^+^·C_7_H_5_ClNO_2_^−^
*M* *_r_* = 376.87
Orthorhombic, 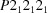

*a* = 10.6756 (3) Å
*b* = 11.5541 (3) Å
*c* = 15.9228 (4) Å
*V* = 1964.03 (9) Å^3^
*Z* = 4
Cu *K*α radiation
μ = 1.90 mm^−1^
*T* = 291 K
0.20 × 0.18 × 0.16 mm

### Data collection  

Agilent Xcalibur Eos Gemini diffractometer
Absorption correction: multi-scan (*CrysAlis PRO*; Agilent, 2012 ▸) *T*_min_ = 0.921, *T*_max_ = 1.000
4968 measured reflections
3173 independent reflections
2805 reflections with *I* > 2σ(*I*)
*R*_int_ = 0.022

### Refinement  

*R*[*F* ^2^ > 2σ(*F* ^2^)] = 0.044
*wR*(*F* ^2^) = 0.121
*S* = 1.03
3173 reflections
239 parameters
H-atom parameters constrained
Δρ_max_ = 0.16 e Å^−3^
Δρ_min_ = −0.18 e Å^−3^
Absolute structure: Flack (1983 ▸), 1001 Friedel pairs
Absolute structure parameter: −0.01 (3)

### 

Data collection: *CrysAlis PRO* (Agilent, 2012 ▸); cell refinement: *CrysAlis PRO*; data reduction: *CrysAlis PRO*; program(s) used to solve structure: *SHELXS97* (Sheldrick, 2008 ▸); program(s) used to refine structure: *SHELXL97* (Sheldrick, 2008 ▸); molecular graphics: *OLEX2* (Dolomanov *et al.*, 2009 ▸); software used to prepare material for publication: *OLEX2*.

## Supplementary Material

Crystal structure: contains datablock(s) I. DOI: 10.1107/S2056989015013389/hb7464sup1.cif

Structure factors: contains datablock(s) I. DOI: 10.1107/S2056989015013389/hb7464Isup2.hkl

Supporting information file. DOI: 10.1107/S2056989015013389/hb7464Isup3.cml

**Fig. 1.**
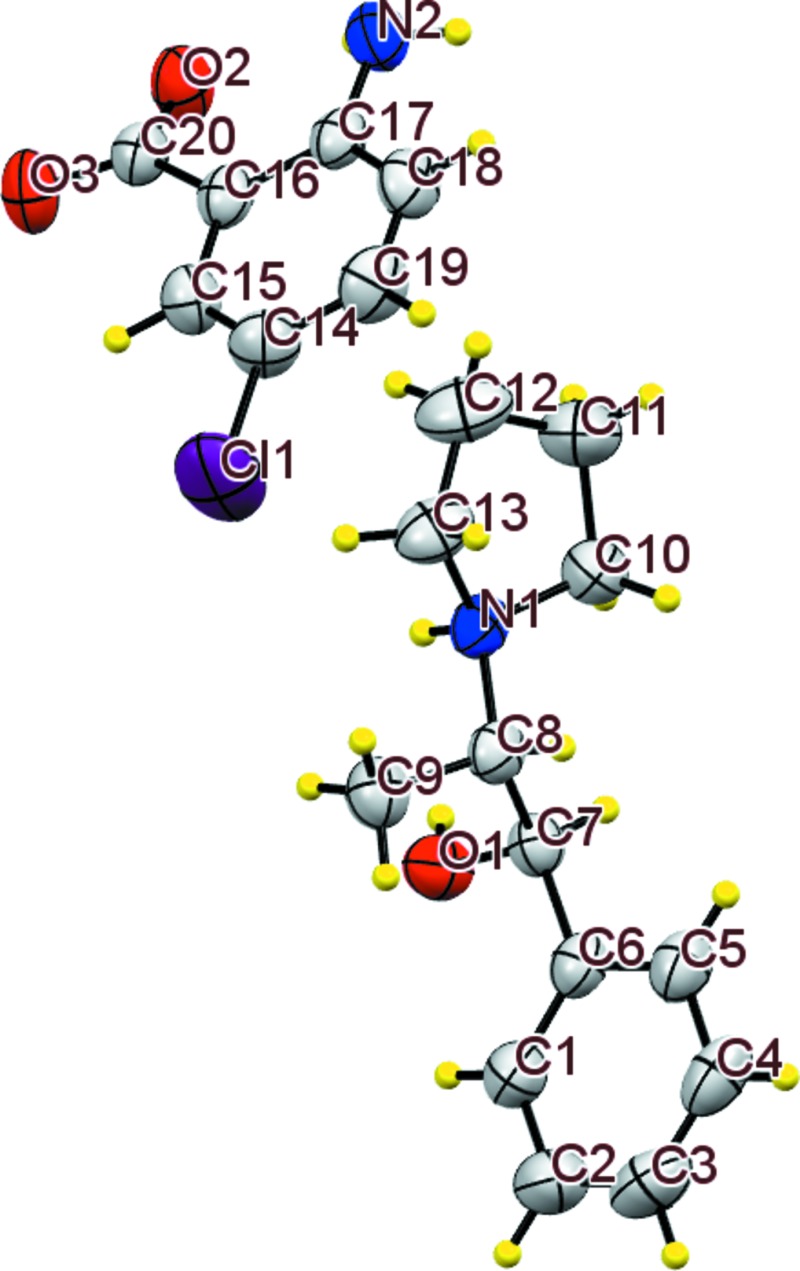
The components of the title salt, showing 50% displacement ellipsoids. Hydrogen bonds are illustrated as dashed lines.

**Fig. 2.**
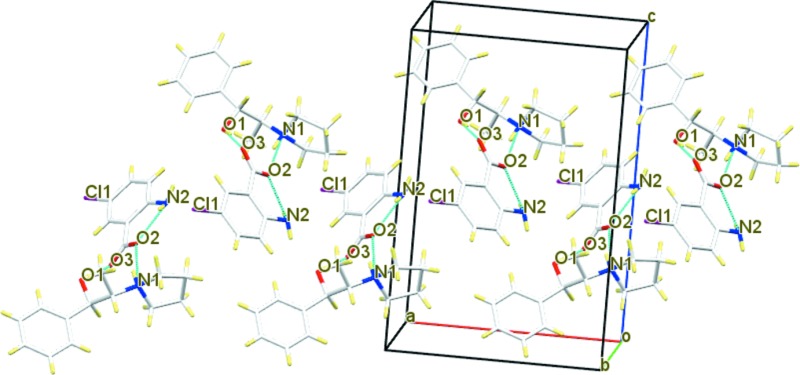
An illustration of the unit cell packing of the title salt viewed down along the b axis. H atoms are omitted for clarity, save those involved in hydrogen bonding.

. DOI: 10.1107/S2056989015013389/hb7464fig1.tif
The components of the title salt, showing 50% displacement ellipsoids. Hydrogen bonds are illustrated as dashed lines.

b . DOI: 10.1107/S2056989015013389/hb7464fig2.tif
An illustration of the unit cell packing of the title salt viewed down along the *b* axis. H atoms are omitted for clarity, save those involved in hydrogen bonding.

CCDC reference: 1044077

Additional supporting information:  crystallographic information; 3D view; checkCIF report

## Figures and Tables

**Table 1:** Hydrogen-bond geometry (, )

*D*H*A*	*D*H	H*A*	*D* *A*	*D*H*A*
O1H1O3^i^	0.82	1.84	2.652(3)	172
N1H1*A*O2^i^	0.91	1.79	2.673(3)	162
N2H2*A*O1^ii^	0.87	2.36	3.137(3)	148
N2H2*B*O2	0.87	2.07	2.686(3)	126

Symmetry codes: (i) 
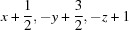
; (ii) 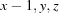.

## supplementary crystallographic information

### S1. Comment

Norephedrine and its derivatives have used widely because of these using for asymmetric synthesis as catalysts or starting materials (Kanizsai *et al.*, 2006); Rzaczynska *et al.* (2000); The crystal structure of (1R,2R)-amino­alcohol·HCl (Pennemann *et al.*, 2000), amino­pyridine (Sugiyama *et al.*, 2002), Morpholinium 5-chloro-2-nitro­benzoate (Ishida *et al.*, 2001) have been reported. To our knowledge, this is the first structural report of a pyrrolidinium system. We here report the crystal structure of the title compound.

In the title compound (Fig.1), the bond lengths and angles are normal (Arora & Pant, 1969). The asymmetric unit contains one cation–anion pair. The molecular packing features an N—H···O hydrogen bond between the amino group and the oxygen of the carbonyl group. In the crystal, the cation and anion are linked by an N—H···O, O—H···O inter­action (Table 1 and Fig.2).

### S2. Experimental

An crystal structure salt in the 1:1.5 ration of the original partners of the 2-amino-5-chloro-benzoic acid (5.0 g, 0.0224 mol) and added a chiral additive (1R,2S)-1-phenyl-2-(1-pyrrolidinyl)propan-1-ol (6.4 g, 0.0312 mol). Under the condition of sodium hydride (1.4 g, 0.0583 mol) alkali, choosing anhydrous THF (30 ml) inert solvent under nitro­gen atmosphere was stirred for 2h and the process was inspected by TLC. At the end of the reaction processing, the pale yellow prismatic crystals were obtained from the solution by slow volatilization from the solvent of the ethyl acetate after at room temperature overnight. (yield 89%, M.pt: 402-404K). ^1^H NMR(400Hz, CDCl_3_): δ(ppm): 3.0~3.5 (m,5H,Ar), 2.0~2.2 (e, 8H, CH_2_), 1.1~1.3 (t,3H,CH_3_), 5.5 (s,1H,OH), 6.5(s,1H,CHOH),7.0 (s,1H,CHCH_3_),7.3 (s,1H,NH), 7.4~7.5(t,3H,Ar), 8.0 (q,2H,NH_2_). ^13^C NMR(100MHz, CDCl_3_): δ(ppm) 9.3, 23.1, 52.5, 68.2, 70.9, 77.5, 117.6, 120.5, 125.7, 127.3, 128.3, 131.8, 140.5, 148.4, 174.9.

### S3. Refinement

The H atoms attached to N atoms were located in a difference Fourier-map analyses and were allowed to ride in the refinements. The C-bound H atoms were positioned geometrically and allowed to ride on their parent atoms, with Uiso(H) = 1.5Ueq(C) for methyl group and Uiso(H)= 1.5Ueq(O) for hydroxyl H atom and equal to 1.2Ueq(C,N) for other H atoms.

### Crystal data

**Table:**

C_13_H_20_NO^+^·C_7_H_5_ClNO_2_^−^	*D*_x_ = 1.275 Mg m^−^^3^
*M**_r_* = 376.87	Melting point = 402–404 K
Orthorhombic, *P*2_1_2_1_2_1_	Cu *K*α radiation, λ = 1.54184 Å
*a* = 10.6756 (3) Å	Cell parameters from 1586 reflections
*b* = 11.5541 (3) Å	θ = 4.7–71.7°
*c* = 15.9228 (4) Å	µ = 1.90 mm^−^^1^
*V* = 1964.03 (9) Å^3^	*T* = 291 K
*Z* = 4	, yellow
*F*(000) = 800	0.20 × 0.18 × 0.16 mm

### Data collection

**Table:**

Agilent Xcalibur Eos Gemini diffractometer	3173 independent reflections
Radiation source: Enhance (Cu) X-ray Source	2805 reflections with *I* > 2σ(*I*)
Graphite monochromator	*R*_int_ = 0.022
Detector resolution: 16.2312 pixels mm^-1^	θ_max_ = 72.3°, θ_min_ = 4.7°
ω scans	*h* = −8→13
Absorption correction: multi-scan (*CrysAlis PRO*; Agilent, 2012)	*k* = −13→8
*T*_min_ = 0.921, *T*_max_ = 1.000	*l* = −19→18
4968 measured reflections	

### Refinement

**Table:**

Refinement on *F*^2^	Hydrogen site location: inferred from neighbouring sites
Least-squares matrix: full	H-atom parameters constrained
*R*[*F*^2^ > 2σ(*F*^2^)] = 0.044	*w* = 1/[σ^2^(*F*_o_^2^) + (0.0583*P*)^2^ + 0.0389*P*] where *P* = (*F*_o_^2^ + 2*F*_c_^2^)/3
*wR*(*F*^2^) = 0.121	(Δ/σ)_max_ = 0.001
*S* = 1.03	Δρ_max_ = 0.16 e Å^−^^3^
3173 reflections	Δρ_min_ = −0.18 e Å^−^^3^
239 parameters	Extinction correction: *SHELXL97* (Sheldrick, 2008), Fc^*^=kFc[1+0.001xFc^2^λ^3^/sin(2θ)]^-1/4^
0 restraints	Extinction coefficient: 0.0019 (4)
Primary atom site location: structure-invariant direct methods	Absolute structure: Flack (1983)
Secondary atom site location: difference Fourier map	Absolute structure parameter: −0.01 (3)

### Special details

**Table:**

Geometry. All e.s.d.'s (except the e.s.d. in the dihedral angle between two l.s. planes) are estimated using the full covariance matrix. The cell e.s.d.'s are taken into account individually in the estimation of e.s.d.'s in distances, angles and torsion angles; correlations between e.s.d.'s in cell parameters are only used when they are defined by crystal symmetry. An approximate (isotropic) treatment of cell e.s.d.'s is used for estimating e.s.d.'s involving l.s. planes.
Refinement. Refinement of *F*^2^ against ALL reflections. The weighted *R*-factor *wR* and goodness of fit *S* are based on *F*^2^, conventional *R*-factors *R* are based on *F*, with *F* set to zero for negative *F*^2^. The threshold expression of *F*^2^ > σ(*F*^2^) is used only for calculating *R*-factors(gt) *etc*. and is not relevant to the choice of reflections for refinement. *R*-factors based on *F*^2^ are statistically about twice as large as those based on *F*, and *R*- factors based on ALL data will be even larger.

### Fractional atomic coordinates and isotropic or equivalent isotropic displacement parameters (Å^2^)

**Table:**

	*x*	*y*	*z*	*U*_iso_*/*U*_eq_	
O1	0.77038 (18)	0.68499 (17)	0.70064 (14)	0.0556 (5)	
H1	0.7330	0.7465	0.6948	0.083*	
N1	0.5009 (2)	0.61324 (19)	0.69466 (13)	0.0457 (5)	
H1A	0.5313	0.6659	0.6574	0.055*	
C1	0.9322 (3)	0.5422 (3)	0.78250 (18)	0.0595 (7)	
H1B	0.9576	0.5879	0.7375	0.071*	
C2	1.0177 (3)	0.4718 (4)	0.8223 (2)	0.0707 (9)	
H2	1.0999	0.4689	0.8032	0.085*	
C3	0.9826 (4)	0.4061 (3)	0.8895 (2)	0.0759 (10)	
H3	1.0408	0.3587	0.9161	0.091*	
C4	0.8608 (4)	0.4099 (3)	0.9180 (2)	0.0692 (9)	
H4	0.8373	0.3655	0.9641	0.083*	
C5	0.7729 (3)	0.4797 (3)	0.87827 (17)	0.0582 (7)	
H5	0.6909	0.4824	0.8978	0.070*	
C6	0.8084 (3)	0.5455 (2)	0.80910 (16)	0.0471 (6)	
C7	0.7116 (2)	0.6191 (2)	0.76352 (15)	0.0446 (5)	
H7	0.6722	0.6716	0.8040	0.054*	
C8	0.6092 (2)	0.5410 (2)	0.72422 (15)	0.0449 (5)	
H8	0.5789	0.4877	0.7676	0.054*	
C9	0.6609 (3)	0.4695 (3)	0.65138 (18)	0.0575 (7)	
H9A	0.5995	0.4135	0.6343	0.086*	
H9B	0.7357	0.4303	0.6690	0.086*	
H9C	0.6799	0.5197	0.6051	0.086*	
C10	0.4349 (3)	0.6792 (3)	0.76326 (19)	0.0570 (7)	
H10A	0.4749	0.7535	0.7723	0.068*	
H10B	0.4369	0.6359	0.8154	0.068*	
C11	0.3014 (3)	0.6954 (4)	0.7337 (3)	0.0786 (10)	
H11A	0.2431	0.6675	0.7759	0.094*	
H11B	0.2844	0.7767	0.7236	0.094*	
C12	0.2876 (3)	0.6270 (4)	0.6535 (3)	0.0823 (11)	
H12A	0.2878	0.6780	0.6052	0.099*	
H12B	0.2099	0.5834	0.6538	0.099*	
C13	0.3990 (3)	0.5462 (3)	0.6511 (2)	0.0619 (8)	
H13A	0.3810	0.4746	0.6805	0.074*	
H13B	0.4224	0.5283	0.5936	0.074*	
Cl1	0.32906 (11)	0.26127 (9)	0.50325 (8)	0.0991 (4)	
O2	0.0402 (2)	0.72036 (18)	0.41891 (13)	0.0626 (6)	
O3	0.1584 (2)	0.61558 (18)	0.33345 (12)	0.0613 (5)	
N2	−0.0763 (2)	0.6051 (2)	0.54289 (16)	0.0574 (6)	
H2A	−0.0982	0.6071	0.5956	0.069*	
H2B	−0.0522	0.6745	0.5286	0.069*	
C14	0.2067 (3)	0.3611 (2)	0.51305 (19)	0.0573 (7)	
C15	0.1973 (3)	0.4508 (2)	0.45717 (17)	0.0504 (6)	
H15	0.2547	0.4565	0.4134	0.060*	
C16	0.1037 (2)	0.5333 (2)	0.46472 (15)	0.0425 (5)	
C17	0.0182 (2)	0.5255 (2)	0.53195 (15)	0.0430 (5)	
C18	0.0277 (3)	0.4295 (3)	0.58599 (18)	0.0540 (6)	
H18	−0.0307	0.4206	0.6288	0.065*	
C19	0.1203 (3)	0.3492 (3)	0.5773 (2)	0.0611 (7)	
H19	0.1254	0.2869	0.6142	0.073*	
C20	0.0996 (2)	0.6300 (2)	0.40106 (15)	0.0458 (5)	

### Atomic displacement parameters (Å^2^)

**Table:**

	*U*^11^	*U*^22^	*U*^33^	*U*^12^	*U*^13^	*U*^23^
O1	0.0505 (9)	0.0523 (10)	0.0639 (12)	−0.0011 (8)	0.0042 (9)	0.0167 (10)
N1	0.0496 (11)	0.0451 (11)	0.0423 (10)	−0.0102 (9)	−0.0022 (9)	0.0083 (9)
C1	0.0569 (14)	0.0726 (18)	0.0491 (14)	0.0000 (15)	−0.0041 (13)	0.0039 (14)
C2	0.0573 (16)	0.088 (2)	0.0673 (19)	0.0102 (18)	−0.0088 (15)	−0.0005 (19)
C3	0.077 (2)	0.075 (2)	0.076 (2)	0.0115 (19)	−0.0265 (19)	0.0090 (18)
C4	0.091 (2)	0.0674 (18)	0.0497 (15)	−0.0090 (18)	−0.0199 (17)	0.0132 (15)
C5	0.0666 (17)	0.0628 (16)	0.0451 (13)	−0.0063 (15)	−0.0043 (13)	0.0053 (13)
C6	0.0533 (13)	0.0483 (12)	0.0398 (11)	−0.0044 (12)	−0.0054 (10)	−0.0022 (10)
C7	0.0466 (12)	0.0443 (12)	0.0430 (11)	−0.0023 (11)	0.0029 (10)	0.0001 (11)
C8	0.0479 (12)	0.0453 (12)	0.0417 (11)	−0.0060 (11)	0.0003 (10)	0.0068 (11)
C9	0.0623 (16)	0.0576 (15)	0.0526 (14)	−0.0035 (14)	0.0025 (13)	−0.0034 (13)
C10	0.0535 (15)	0.0634 (16)	0.0541 (15)	−0.0003 (14)	0.0004 (13)	−0.0008 (13)
C11	0.0552 (17)	0.098 (3)	0.083 (2)	0.0080 (18)	−0.0053 (17)	0.003 (2)
C12	0.0629 (19)	0.076 (2)	0.108 (3)	−0.0098 (19)	−0.029 (2)	−0.003 (2)
C13	0.0595 (16)	0.0545 (16)	0.0716 (18)	−0.0127 (14)	−0.0177 (15)	−0.0005 (15)
Cl1	0.1099 (8)	0.0790 (6)	0.1084 (8)	0.0480 (6)	0.0236 (7)	0.0237 (6)
O2	0.0816 (14)	0.0535 (11)	0.0528 (11)	0.0145 (11)	0.0142 (11)	0.0138 (9)
O3	0.0859 (15)	0.0529 (11)	0.0452 (9)	0.0028 (11)	0.0154 (10)	0.0043 (9)
N2	0.0577 (13)	0.0626 (14)	0.0520 (12)	0.0023 (12)	0.0097 (11)	0.0053 (11)
C14	0.0618 (16)	0.0483 (13)	0.0617 (16)	0.0100 (13)	−0.0003 (14)	0.0026 (13)
C15	0.0571 (14)	0.0473 (13)	0.0468 (12)	−0.0003 (12)	0.0056 (12)	−0.0012 (11)
C16	0.0482 (12)	0.0411 (11)	0.0383 (10)	−0.0063 (10)	−0.0027 (10)	−0.0040 (10)
C17	0.0448 (11)	0.0446 (12)	0.0397 (11)	−0.0069 (11)	−0.0053 (10)	−0.0005 (10)
C18	0.0546 (14)	0.0562 (15)	0.0511 (14)	−0.0126 (13)	0.0054 (13)	0.0078 (13)
C19	0.0754 (19)	0.0474 (14)	0.0607 (16)	−0.0029 (14)	−0.0009 (16)	0.0147 (13)
C20	0.0508 (13)	0.0468 (13)	0.0398 (12)	−0.0027 (11)	−0.0003 (10)	0.0012 (10)

### Geometric parameters (Å, º)

**Table:**

O1—H1	0.8200	C10—H10B	0.9700
O1—C7	1.406 (3)	C10—C11	1.513 (4)
N1—H1A	0.9100	C11—H11A	0.9700
N1—C8	1.502 (3)	C11—H11B	0.9700
N1—C10	1.506 (4)	C11—C12	1.509 (5)
N1—C13	1.505 (3)	C12—H12A	0.9700
C1—H1B	0.9300	C12—H12B	0.9700
C1—C2	1.377 (5)	C12—C13	1.512 (5)
C1—C6	1.389 (4)	C13—H13A	0.9700
C2—H2	0.9300	C13—H13B	0.9700
C2—C3	1.365 (5)	Cl1—C14	1.750 (3)
C3—H3	0.9300	O2—C20	1.254 (3)
C3—C4	1.377 (6)	O3—C20	1.257 (3)
C4—H4	0.9300	N2—H2A	0.8710
C4—C5	1.389 (5)	N2—H2B	0.8724
C5—H5	0.9300	N2—C17	1.376 (3)
C5—C6	1.391 (4)	C14—C15	1.369 (4)
C6—C7	1.522 (4)	C14—C19	1.384 (4)
C7—H7	0.9800	C15—H15	0.9300
C7—C8	1.549 (3)	C15—C16	1.386 (4)
C8—H8	0.9800	C16—C17	1.410 (4)
C8—C9	1.527 (4)	C16—C20	1.510 (4)
C9—H9A	0.9600	C17—C18	1.408 (4)
C9—H9B	0.9600	C18—H18	0.9300
C9—H9C	0.9600	C18—C19	1.363 (4)
C10—H10A	0.9700	C19—H19	0.9300
			
C7—O1—H1	109.5	H10A—C10—H10B	108.7
C8—N1—H1A	107.5	C11—C10—H10A	110.5
C8—N1—C10	114.4 (2)	C11—C10—H10B	110.5
C8—N1—C13	114.5 (2)	C10—C11—H11A	110.4
C10—N1—H1A	107.5	C10—C11—H11B	110.4
C13—N1—H1A	107.5	H11A—C11—H11B	108.6
C13—N1—C10	104.9 (2)	C12—C11—C10	106.9 (3)
C2—C1—H1B	119.8	C12—C11—H11A	110.4
C2—C1—C6	120.4 (3)	C12—C11—H11B	110.4
C6—C1—H1B	119.8	C11—C12—H12A	110.6
C1—C2—H2	119.7	C11—C12—H12B	110.6
C3—C2—C1	120.5 (3)	C11—C12—C13	105.6 (3)
C3—C2—H2	119.7	H12A—C12—H12B	108.8
C2—C3—H3	120.0	C13—C12—H12A	110.6
C2—C3—C4	119.9 (3)	C13—C12—H12B	110.6
C4—C3—H3	120.0	N1—C13—C12	103.8 (2)
C3—C4—H4	119.8	N1—C13—H13A	111.0
C3—C4—C5	120.4 (3)	N1—C13—H13B	111.0
C5—C4—H4	119.8	C12—C13—H13A	111.0
C4—C5—H5	120.2	C12—C13—H13B	111.0
C4—C5—C6	119.6 (3)	H13A—C13—H13B	109.0
C6—C5—H5	120.2	H2A—N2—H2B	107.7
C1—C6—C5	119.0 (3)	C17—N2—H2A	109.7
C1—C6—C7	121.1 (2)	C17—N2—H2B	111.5
C5—C6—C7	119.9 (3)	C15—C14—Cl1	119.7 (2)
O1—C7—C6	109.8 (2)	C15—C14—C19	120.5 (3)
O1—C7—H7	108.9	C19—C14—Cl1	119.8 (2)
O1—C7—C8	110.0 (2)	C14—C15—H15	119.5
C6—C7—H7	108.9	C14—C15—C16	121.1 (3)
C6—C7—C8	110.3 (2)	C16—C15—H15	119.5
C8—C7—H7	108.9	C15—C16—C17	119.3 (2)
N1—C8—C7	110.2 (2)	C15—C16—C20	118.1 (2)
N1—C8—H8	108.3	C17—C16—C20	122.6 (2)
N1—C8—C9	109.9 (2)	N2—C17—C16	121.9 (2)
C7—C8—H8	108.3	N2—C17—C18	120.2 (2)
C9—C8—C7	111.5 (2)	C18—C17—C16	117.9 (2)
C9—C8—H8	108.3	C17—C18—H18	119.1
C8—C9—H9A	109.5	C19—C18—C17	121.8 (3)
C8—C9—H9B	109.5	C19—C18—H18	119.1
C8—C9—H9C	109.5	C14—C19—H19	120.3
H9A—C9—H9B	109.5	C18—C19—C14	119.3 (3)
H9A—C9—H9C	109.5	C18—C19—H19	120.3
H9B—C9—H9C	109.5	O2—C20—O3	123.9 (2)
N1—C10—H10A	110.5	O2—C20—C16	118.6 (2)
N1—C10—H10B	110.5	O3—C20—C16	117.6 (2)
N1—C10—C11	106.1 (3)		
			
O1—C7—C8—N1	−70.2 (3)	C10—C11—C12—C13	15.7 (4)
O1—C7—C8—C9	52.3 (3)	C11—C12—C13—N1	−31.8 (4)
N1—C10—C11—C12	6.5 (4)	C13—N1—C8—C7	176.8 (2)
C1—C2—C3—C4	0.1 (6)	C13—N1—C8—C9	53.5 (3)
C1—C6—C7—O1	−5.3 (4)	C13—N1—C10—C11	−26.4 (3)
C1—C6—C7—C8	116.1 (3)	Cl1—C14—C15—C16	−178.1 (2)
C2—C1—C6—C5	2.2 (5)	Cl1—C14—C19—C18	177.9 (2)
C2—C1—C6—C7	−177.4 (3)	N2—C17—C18—C19	−179.7 (3)
C2—C3—C4—C5	0.5 (6)	C14—C15—C16—C17	1.1 (4)
C3—C4—C5—C6	0.3 (5)	C14—C15—C16—C20	179.7 (3)
C4—C5—C6—C1	−1.6 (4)	C15—C14—C19—C18	−1.9 (5)
C4—C5—C6—C7	178.0 (3)	C15—C16—C17—N2	179.7 (2)
C5—C6—C7—O1	175.1 (2)	C15—C16—C17—C18	−3.6 (4)
C5—C6—C7—C8	−63.5 (3)	C15—C16—C20—O2	−160.7 (3)
C6—C1—C2—C3	−1.4 (6)	C15—C16—C20—O3	18.1 (4)
C6—C7—C8—N1	168.5 (2)	C16—C17—C18—C19	3.5 (4)
C6—C7—C8—C9	−69.0 (3)	C17—C16—C20—O2	17.8 (4)
C8—N1—C10—C11	−152.7 (3)	C17—C16—C20—O3	−163.4 (2)
C8—N1—C13—C12	162.2 (3)	C17—C18—C19—C14	−0.8 (5)
C10—N1—C8—C7	−62.0 (3)	C19—C14—C15—C16	1.7 (5)
C10—N1—C8—C9	174.6 (2)	C20—C16—C17—N2	1.2 (4)
C10—N1—C13—C12	35.9 (3)	C20—C16—C17—C18	177.9 (2)

### Hydrogen-bond geometry (Å, º)

**Table:**

*D*—H···*A*	*D*—H	H···*A*	*D*···*A*	*D*—H···*A*
O1—H1···O3^i^	0.82	1.84	2.652 (3)	172
N1—H1*A*···O2^i^	0.91	1.79	2.673 (3)	162
N2—H2*A*···O1^ii^	0.87	2.36	3.137 (3)	148
N2—H2*B*···O2	0.87	2.07	2.686 (3)	126

Symmetry codes: (i) *x*+1/2, −*y*+3/2, −*z*+1; (ii) *x*−1, *y*, *z*.
