# Supplementary figures and images for: Crystal structure of 1-[(1S,2R)-2-hydroxy-1-methyl-2-phenyl­eth­yl]pyrrolidinium 2-amino-5-chloro­benzoate
Source: Acta Crystallogr E Crystallogr Commun. 2015 Jul 17;71(Pt 8):o584–5. doi: 10.1107/S2056989015013389 (PMC4571409; doi:10.1107/S2056989015013389)

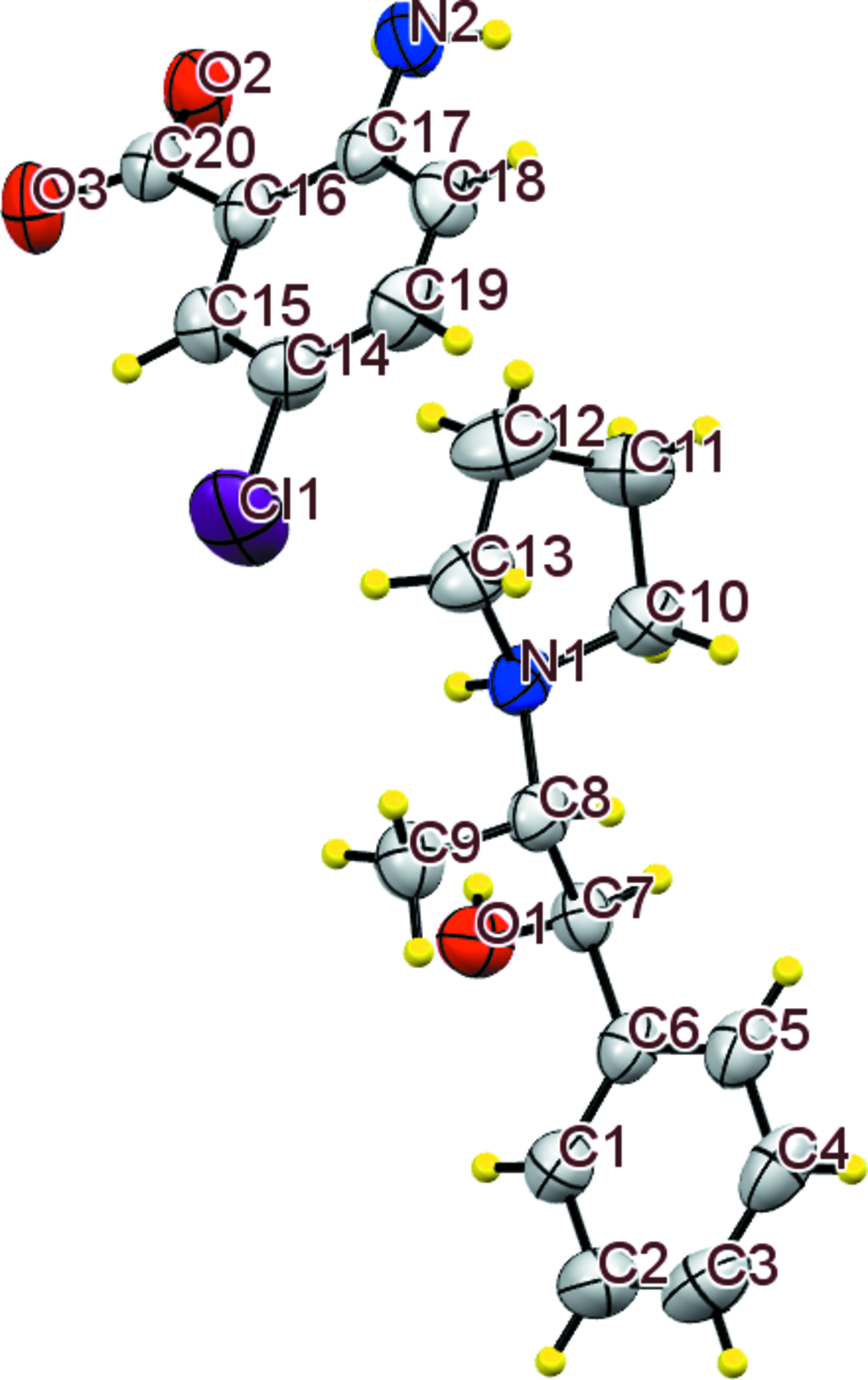

Supplement: Supplementary file 4 [file e-71-0o584-fig1.tif]

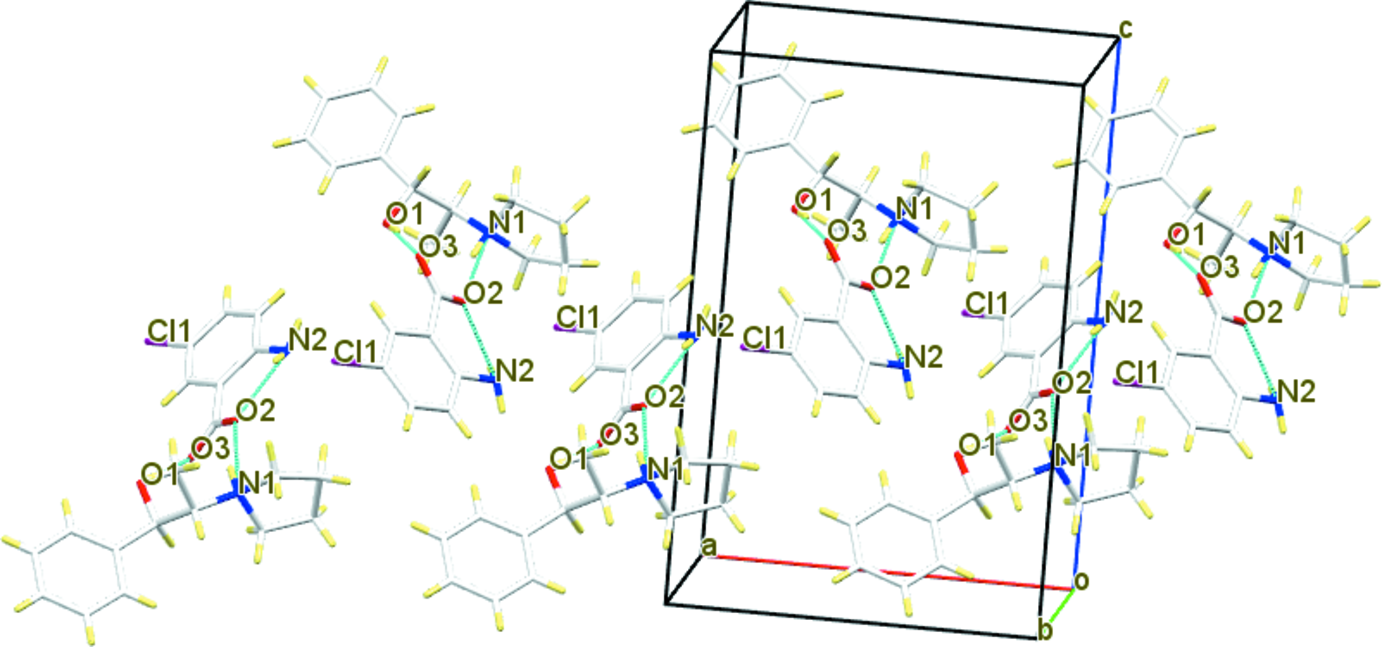

Supplement: Supplementary file 5 [file e-71-0o584-fig2.tif]
